# Emergence of multidrug resistant, *ctx* negative seventh pandemic *Vibrio cholerae* O1 El Tor sequence type (ST) 69 in coastal water of Kerala, India

**DOI:** 10.1038/s41598-023-50536-z

**Published:** 2024-01-23

**Authors:** Minimol V. Ayyappan, Pankaj Kishore, Satyen Kumar Panda, Anuj Kumar, Devananda Uchoi, Ranjit Kumar Nadella, Himanshu Priyadarshi, Mohan Chitradurga Obaiah, Dybin George, Muneeb Hamza, Sreelekshmi K. Ramannathan, C. N. Ravishankar

**Affiliations:** 1 ICAR-Central Institute of Fisheries Technology, Kochi, India; 2 Food Safety and Standard Authority of India, New Delhi, India; 3 ICAR–Indian Institute of Wheat and Barley Research, Karnal, Haryana India; 4 College of Fisheries, Lembucherra, Tripura India; 5 Kerala University of Fisheries and Ocean Studies, Kochi, India; 6 Cochin University of Science and Technology, Kochi, India; 7 ICAR-Central Institute of Fisheries Education, Mumbai, India

**Keywords:** Microbiology, Environmental microbiology

## Abstract

Seventh pandemic *Vibrio choleare* O1 El Tor strain is responsible for the on-going pandemic outbreak of cholera globally. This strain evolved from non-pathogenic *V. cholerae* by acquiring seventh pandemic gene (VC 2346), pandemic Islands (VSP1 and VSP2), pathogenicity islands (VP1 and VP2) and CTX prophage region. The cholera toxin production is mainly attributed to the presence of *ctx* gene in these strains. However, several variants of this strain emerged as hybrid strains or atypical strains. The present study aimed to assess the aquatic environment of Cochin, India, over a period of 5 years for the emergence of multidrug resistant *V. cholerae* and its similarity with seventh pandemic strain. The continuous surveillance and monitoring resulted in the isolation of *ctx* negative, O1 positive *V. cholerae* isolate (VC6) from coastal water, Cochin, Kerala. The isolate possessed the biotype specific O1 El Tor *tcpA* gene and lacked other biotype specific *ctx, zot, ace* and *rst* genes. Whole genome analysis revealed the isolate belongs to pandemic sequence type (ST) 69 with the possession of pandemic VC2346 gene, pathogenic island VPI1, VPI2, and pandemic island VSP1 and VSP2. The isolate possessed several insertion sequences and the SXT/R391 family related Integrative Conjugative Elements (ICEs)*.* In addition to this, the isolate genome carried virulence genes such as *VgrG, mshA, ompT, toxR, ompU, rtxA*, *als*, *VasX, makA*, and *hlyA* and antimicrobial resistance genes such as *gyrA, dfrA1, strB, parE, sul2, parC, strA*, VC1786ICE9-*floR,* and *catB9.* Moreover, the phylogenetic analysis suggests that the isolate genome is more closely related to seventh pandemic *V.* *cholerae* O1 N16961 strain. This study reports the first incidence of environmental *ctx* negative seventh pandemic *V. choleare* O1 El Tor isolate, globally and its presence in the aquatic system likely to induce toxicity in terms of public health point of view. The presence of this isolate in the aquatic environment warns the strict implementation of the epidemiological surveillance on the occurrence of emerging strains and the execution of flagship program for the judicious use of antibiotics in the aquatic ecosystem.

## Introduction

The increased rate of antimicrobial resistance and rising temperature highlights the importance of surveillance of various human pathogens of both emerging and reemerging category. *Vibrio cholerae,* a well-known human pathogen of pandemic potential, is widely distributed in aquatic environment especially in brackish water ecosystem^1^. Among the 200 serogroups of this pathogen^22^, pandemic *V. cholerae* O1 serogroup, in which classical biotype had been responsible for cholera outbreaks in Nineteenth Century later it was substituted by EI Tor biotype in the Twentieth Century^3^. The seventh pandemics in cholera outbreak started in the year 1961 in Indonesian Island and spread gradually to other continents such as Asia, Africa and America^4^. The emergence of sero and bio variants of O1 strains have been reported there after in several parts of the world^5–7^. Pandemic *V. cholerae* O1 biotype known to carry pandemic islands (VSP1 and VSP2) and pathogenicity islands (VPI1 and VPI2) together with *ctx* genetic element comprising core and RS2 regions^8^. The major virulence factor lies in the core region which encode *ctx* and other accessory toxin genes required for the infectious cholera diseases^9^. The seventh pandemic O1 El Tor strains have been categorized into three independent waves such as wave 1, wave 2, wave 3 based on their evolutionary analysis via SNP, and variations in *ctx* and phage repressor gene (*rstR*) genes^34^. The mobile genetic elements root the acquisition of virulence and AMR genes^35^. Subsequently, several atypical/variant/hybrid strains of O1 El Tor which are known to carry *tcpA* classical genes or classical type of *ctxB* gene have been reported from clinical cases^45^. Similarly, O1 EI Tor, *ctx* negative, non-pandemic strains have been reported from both clinical and environmental sources from South America, and Australia^36,37^. The mechanism of virulence and pathogenicity of this strain remains unknown. Recently, O1 EI Tor positive, *ctx* negative and non-pandemic strain termed as non-toxigenic strain was reported in Haitian aquatic environments with an unknown source of evolutionary origin^38^. This causes a fundamental question of the already known facts of the origin of toxigenic O1 through lateral gene transfer of the CTXφ in the history of epidemic cholera. Most of the seventh pandemic strains belongs to the sequence type ST69 and rarely to ST515^23,24^ and interestingly, none of the previously reported environmental strains of *V. cholerae* belonged to these sequence type.

The present study was undertaken to monitor the aquatic environment of Cochin, Kerala, India for the presence of new variants of *V. cholerae* for a period of 05 years (2018–2022)*.* The study revealed an unusual molecular feature of *ctx* negative O1 positive characteristics in one isolate from coastal water near to a retail seafood market of Thoppumpady fishing harbour, Cochin. This unusual molecular characteristic of the isolate prompted us to investigate further considering the significance of this pathogen to public health. In the present study, we report the presence of new strain of *V. cholerae* O1 El Tor lacking *ctx, zot* and *ace* gene and attribute to the pandemic sequence type 69 by possessing pandemic features and multidrug antimicrobial resistant pattern. The phenotypic and genotypic characteristics of the isolate are presented here by employing conventional microbiological testing, slide agglutination, virulence characterization, whole genome comparison to ascertain the implications of the evolutionary existence of the emerging strain in the aquatic environment. However, O1 EI Tor positive, *ctx* negative with pandemic characteristics belonging to a pandemic sequence type with the possession of SXT/R391 family related ICEs elements have not been reported previously from both clinical as well as from environmental sources. The presence of this novel isolate in aquatic environment marked the importance of this reservoir in the emergence and evolutionary dynamics of *V. cholerae.*

## Materials and methods

### Isolation and identification of V. cholerae

A total of 1237 samples comprising fish, shellfish, water, and sediments from fish markets, landing centres, coastal and aquaculture environments, were screened for the presence of *V. cholerae* over a period of five years (2018–2022)*.* Samples were brought to the Microbiology laboratory of ICAR- Central Institute of Fisheries Technology, Cochin in sterile sample collection bags in iced condition (0–2 °C). Isolation and biochemical identification of *V*. c*holerae* from different samples was carried out as per Bacteriological Analytical Manual protocol for *Vibrios*^39^. Identification of the isolates were confirmed by serogroup test using growth from triple sugar iron agar slant with polyvalent O1 and monospecific Inaba and Ogawa anti-sera (Becton Dickinson, USA). All the isolates were maintained in tryptic soy broth (Difco, USA) supplemented with 30% glycerol at − 80 °C or in Luria–Bertani agar stab culture at room temperature. Molecular identification with respect to virulence, and antigenic types was carried out by polymerase chain reaction (PCR) targeting species specific *ompW* gene^2^, serotype specific *rfb*-O1, *rfb*-O139 gene^40^, biotype specific *tcpA* (classical), and *tcpA* (El Tor) genes^40^, and virulence specific *toxR, hlyA, ctx, zot,* and *ace* genes^40–42^. Details of primers and PCR conditions used in this study are listed in Table 1.

**Table 1:** Details of primers and PCR conditions used in this study.

Primers	Sequence (5’-3’)	PCR Condition	Amplicon size (bp)
*ompW*	f-CACCAAGAAGGTGACTTTATTGTG r-GAACTTATAACCACCCGCG	94 °C for 30 s, 64 °C for 30 s, 72 °C for 30 s	586
*rfb*-O1	f-GTTTCACTGAACAGATGGG r-GGTCATCTGTAAGTACAAC	94 °C for 1 min, 55 °C for 1 min, 72 °C for 1 min	192
*rfb*-O139	f-AGCCTCTTTATTACGGGTGG r-GTCAAACCCGATCGTAAAGG	94 °C for 1 min, 55 °C for 1 min, 72 °C for 1 min	449
*tcpA* (Classica)	f-CACGATAGGAAAACCGGTCAAGAG r-ACCAAATGCAACGCCGAATCGAG	94 °C for 1 min, 60 °C for 1 min, 72 °C for 1 min	617
*tcpA* (El Tor)	f-AAGAAGTTTGTAAAAGAAGAACAC r-GAAAGGACCTTCTTTCACGTTG	94 °C for 1 min, 60 °C for 1 min, 72 °C for 1 min	471
*toxR,*	f-TGTTCGGATTAGGACAC r-TACTCACACACTTTGATGGC	94 °C for 1 min, 60 °C for 1 min, 72 °C for 1 min	883
*hlyA*	f-GGC AAA CAG CGA AAC AAA TAC C r-CTC AGC GGG CTA ATA CGG TTT A	94 °C for 1 min, 60 °C for 1 min, 72 °C for 1 min	481
*ctx*	f-CGGGCAGATTCTAGACCTCCTG r-CGATGATCTTGGAGCATTCCCAC	94 °C for 1 min, 60 °C for 1 min, 72 °C for 1 min	564
*zot*	f-TCGCTTAACGATGGCGCGTTTT r-AACCCCGTTTCACTTCTACCCA	94 °C for 1 min, 60 °C for 1 min, 72 °C for 1 min	243
*ace*	f-GCTTATGATGGACACCCTTTA r-TTTGCCCTGCGAGCGTTAAAC	94 °C for 1 min, 55 °C for 1 min, 72 °C for 1 min	284

### Whole genome sequencing, gene assembly and annotation

The bacterial DNA was extracted from overnight culture using Qiagen DNA extraction kit (Qiagen, Germany) according to the manufacturer’s instructions. The quality of the DNA was checked using a nanodrop (Thermo Scientific, USA). A ratio of OD_260/280_ value of 1.8–1.9 is taken for sequencing. The total DNA was sequenced using Illumina HiSeq2500 sequencing platform and the next generation sequencing library preparation were constructed following manufacturers instruction. (NEBNext® Ultra™II DNA Library Prep Kit for Illumina®, Illumina, San Diego, CA, USA). Sequencing was carried out using a 2X 150 paired end configuration, image analysis and base calling were conducted by HiSeq Control Software (HCS) + OLB + GA pipeline-1.6 (Illumina) on the HiSeq Instrument HiSeq2500.

The quality of the reads obtained from FASTQC tool (https://www.bioinformatics.babraham.ac.uk/projects/fastqc/) were checked for base quality score distribution, sequence quality score distribution, average base content per read, GC distribution in the reads, PCR amplification issue, and presence of over-represented sequences. The low-quality bases were trimmed using custom made perl script, and the adapter sequences were further removed from the reads using cutadapt (version 2.0) (Martin 2011). The trimmed reads were then assembled using SPAdes v 1.13.4 and the quality of the assembled reads were examined using quality assessment tool (QUAST). Annotation was done by RAST tool kit (RASTtk)^49^ available in PATRIC (Pathosystems Resource Integration Center), the bacterial bioinformatics database and analysis resource (http://www.patricbrc.org) using the genetic code 11 and assigned a unique genome identifier of 135623.180^48^.

### Downstream analysis of whole genome data

The genomic contigs in FASTA format were analysed using web based open access tools available at Center for Genomic Epidemiology (CGE) website. Bacterial species was identified by k-mer finder 3.2 (https://cge.cbs.dtu.dk//services/k-merFinder/). Virulence, integrative conjugative elements, and antimicrobial resistance were identified using cholera finder 1.0 (https://cge.cbs.dtu.dk//services/choleraFinder/) with the 98% threshold for identity and with 60% of minimum coverage length, and comprehensive genome analysis tool using PATRIC (Pathosystems Resource Integration Center), the bacterial bioinformatics database and analysis resource (http://www.patricbrc.org). Antimicrobial resistance genes were also identified using ResFinder 4.1 (https://cge.cbs.dtu.dk/services/ResFinder/) and resistance gene identifier (RGI) available in comprehensive antimicrobial resistance database (CARD) (https://card.mcmaster.ca/analyze/rgi). The pathogenicity of the isolates against the human host was predicted using Pathogen Finder 1.1 (https://cge.cbs.dtu.dk/services/PathogenFinder/). The mobile genetic element was identified by Mobile Element Finder (https://cge.cbs.dtu.dk/services/MobileElementFinder/). ICE element of the isolates were aligned using mauve sequence alignment using MegaAlign Pro (DNASTAR lnc, USA) software with ICE elements of the reference *V. cholerae* predicted in cholerafinder1.1. Reference ICE used were *V. cholerae* Ind5 integrating conjugative element ICE*Vch*ind5, complete sequence (GQ463142.1), *V. cholerae* strain VC1786ICE genomic sequence (JN648379.1). ICEberg 2.0 was used to find out the putative ICE of the isolate genome and the reference ICE elements (http://dp-mml.sjtu.edu.cn/ICEberg/ICEfinder). Muli Locus Sequence Type (MLST) was determined using MLST 2.0 (https://cge.cbs.dtu.dk//services/MLST/) and pubMLST database (pubmlst.org).

### Comparative genomics

The isolate VC6 was aligned and mapped to reference genomes using mauve multiple genome alignment using MegaAlign Pro software (DNASTAR, lnc, USA). The reference genomes were selected based on the percentage identity shown in k-mer, virulence, pathogen prediction as well as MLST tools. The genome comparison between the isolate and the reference genomes were visualized using BLAST Ring image Generator (BRIG) software^43^.

### Phylogenetic analysis

Reference genome sequences were aligned with isolate genome sequence in MEGA11 software. HKY nucleotide substitution model with default parameters followed by strict clock model and random starting tree were used for the generation of beast file in BEAUti v1.10.4. The evolutionary history of the aligned sequences in beast xml file were assessed by Bayesian phylogenetic analysis using BEAST package v1.10.4. and phylogenetic tree was visualized using Fig Tree v1.4.4. CSIPhylogeny Version 1.4. https://cge.cbs.dtu.dk/services/CSIPhylogeny/) was used for the SNP based phylogenetic analysis. The input parameter used were default parameters specified in the tool with exclusion of heterozygous SNPs. *V. cholerae* O1 El Tor strain N16961 (Accession No: AE003852.1/AE003853.1) genome was used as reference genome. The other closely related genomes available in public databases were selected based on percentage identity found in PATRIC service tool “Similar Genome Finder” (release 3.6.5) in k-mer, virulence, pathogen prediction as well as MLST tools available in CGE website. SNP derived tree was visualized using Fig Tree V1.4.4. Phylogenetic analysis based on PATRIC global protein families were also carried out to determine the phylogenetic placement of the isolate genome. The closest reference and representative genomes were identified by Mash/MinHash method^46^. The protein sequences from these families were aligned with MUSCLE^10^ and the nucleotides for each of those sequences were mapped to the protein alignment. Data matrix formed were analyzed by RaxML with fast bootstrapping.

## Results

### Prevalence of V. cholerae

The overall prevalence of *V. cholerae* was around 23.3%. A total of 2.9% of the isolates belonged to O1 serogroup. Among the O1 isolates, two isolates (VC5 and VC6) recovered from coastal water near to Thoppumpady fishing harbor, Kerala, India (Latitude: 9.93839; Longitude: 76.26066, Year of isolation: October 2019). These isolates were Gram negative, rod shaped, oxidase positive, motile, showed growth in 0 and 3% NaCl. Biochemical characteristics of the isolate is given in Table 2. Serotyping confirmed the isolates belongs to *V. cholerae* O1 Ogawa strain. Molecular test by PCR confirmed isolate’s identity to *V. cholerae* O1 El Tor biotype. PCR test for virulence gene revealed that the isolates lack the presence of *ctx, zot* and *ace* genes. Isolates were positive for *hly* gene. Since the biochemical and molecular characteristics of these two isolates were same, we selected only VC6 for the detailed genomic analysis.

**Table 2:** Biochemical characteristics of the isolate (VC6).

Sl. No	Biochemical test	Result
1	Gram staining	Negative
2	Motility	Motile
3	Oxidase	Positive
4	Lysine decarboxylase test	Positive
5	Ornithine decarboxylase test	Positive
6	Arginine decarboxylase test	Positive
7	Adonitol fermentation test	Negative
8	Arabinose fermentation test	Negative
9	Cellobiose fermentation test	Negative
10	Myo-inositol fermentation test	Negative
11	Lactose fermentation test	Negative
12	Salicin fermentation test	Negative
13	Dextrose fermentation test	Positive
14	d-Galactose fermentation test	Positive
15	maltose fermentation test	Positive
16	d-Mannitol fermentation test	Positive
17	d-Mannose fermentation test	Positive
18	Sucrose fermentation test	Positive
19	Trehalose fermentation test	Positive
20	Indole production	Positive
21	Growth in 0% NaCl	Positive
22	Growth in 3% NaCl	Positive

### Whole genome analysis

Assembled and annotated contigs of the isolate showed good genome quality, annotation statistics and genome statistics. Comprehensive genome analysis service of PATRIC database showed that the assembled genome consists of 75 contigs with the total length of 4,018,937 bp and an average G + C content of 47.49% (supplementary table 1).

### Identification of relevant genes in V. cholerae isolate

The isolate (VC 6) genome was subjected to downstream analyses using various online tools to identify the distribution of various genes. The K-mer finder-3.20 predicted the isolate identity to *V. cholerae* V060002DNA, complete genome with total score 133618 (Acession No:NZ-AP018677.1) with template coverage of 98.88% and p-vale of 1.0e−26. The bacterial similar finder genome tool for estimating genome distance by Mash/MinHash method showed a k-mer count of 969/1000 with *V. choleare* O1 biovar El Tor strain N16961 (Accession No: AE003852.1/AE003853.1). The isolate was predicted as potential human pathogen with a 100% minimum identity threshold and predicted the probability of a human pathogen by 0.834 with matched pathogenic families 715. The downstream analyses revealed that the isolates possess species specific *ompW* gene, serogroup specific *rfb-*O1 gene, biotype specific *tcpA*_et3_*,* and pandemic specific VC2346 gene. The inadequacy of wave determination of the isolates confirmed that the isolates to be of new variant. The pathogenicity and pandemicity of the isolate were confirmed by the presence of pathogenicity island VPI1 and VPI2, and pandemic island VSP1 and VSP2 in the genome of the isolate VC6. The presence of *tcpA*_et3_ biotype specific cholera gene predicted the phenotype of toxin coregulated pilus specific for El Tor and El Tor variant biotypes with 100% identity with *tcpA* gene of *V. cholerae* strain N16961 strain (Accession No: AF325734.1). In addition to toxin co-regulated pilus biosynthesis protein H, the pathogenicity islands of VPI1 also showed the genes for predicted phenotypes of aldehyde dehydrogenase, accessory colonization factor, *acfB,* and integrase protein of phage family with 100% identity with *V. cholerae* strain N16961 strain (Accession No: AE003852.1). The predicted phenotype in pathogenicity Island VPI2 were integrase phage family, helicase (putative), type I restriction enzyme HSdR (putative), N- acetylneuraminate lyase (putative), N-acetylglucosamine-6-phospate deacetylase, Transposase *orfAB*, and transcriptional regulator (putative) with 100% identity with *V. cholerae* strain N16961 strain (Accession No: AE003852.1). The gene identified in pathogenicity island 1 (VC0819, VC0827, and VC0847), pathogenicity Island 2 (VC1873, VC 1776, VC1758, VC1790, VC1765, VC1809, VC1760) and pandemic island 1 (VC0175, VC0178, VC0180, VC0183, VC0185) were shown 100% identity with *V. cholerae* strain N16961 strain (Accession No: AE003852.1). However, VC0180 gene of pandemic island 1 showed only 99.94% similarity for predicting the phenotype *thif* domain-containing protein of *V. cholerae* seventh pandemic island 1 (VSPI) of *V. cholerae* strain N16961 strain (Accession No: AE003852.1). The gene identified in VSP2 are VC0502, VC0514, VC0498, VC0840, VC0512, VC0490, VC0504, and VC0493. All genes in VSP2 were 100% identity with predicted phenotypes of the respective genes such as ribonuclease HI (putative), type IV pilin (putative), methyl accepting chemotaxis protein, phage integrase and other uncharacterized proteins of VSP2. The presence of 7th pandemic VC2346 gene predicted the phenotype of 7th pandemic *V. cholerae* strain specific gene VC2346 of *V. cholerae* O1 biovar El Tor strain N16961 with 100% identity. Virulomes analysis showed the isolates carries the common virulence genes such as *VgrG, mshA, ompT, toxR, ompU, rtxA, als, VasX, makA,* and *hlyA*. However, cytotoxigenic genes such as *ctx, ace, zot, rstR* genes were not identified in the isolate genome. Interestingly no hits were found for phage susceptibility elements confirmed the absence of cholerae prophage in the genomes of the isolate.

Distribution of antimicrobial genes showed 100% identity of sulphonamid resistant *sul2*, trimethoprim resistant *dfrA1*, and streptomycin resistant *strA, strB* genes of SXT constin ICE*Vch*Hai1. Isolates also carries quinolone resistant *parC* gene with 100% identity of *V. cholerae* H1 topoisomerase IV subunit A (*parC*) gene, Accession No: KJ596550.1 and *parE* gene with 99.72% identity *V. cholerae* O139 strain 404 topoisomerase subunit B *(parE)* gene, Accession No GQ502316. In addition to this, the isolate also carries Amphenicol resistant *catB9* gene (100% identity with *Vibrio cholerae* super-integron chloramphenicol acetyltransferase (*catB9*) gene (Accession no: AF462019.1) and *flor* gene with 98.35% identity with *Salmonella typhimurium* DT104 putative efflux protein *flor* gene (Accession No: AF118107.1). Resistance Gene Identifier (RGI) results of CARD shown perfect hits for 5 AMR genes *catB9* (phenicol antibiotics), *almG* and *almF*, (peptide antibiotics), *varG* (carbapenem), *sul2* (sulphonamide antibiotics) genes and strict hits for *strA* and *strB* genes (aminoglycoside antibiotics), *Escherichia coli* EF-Tu mutants conferring resistance to Pulvomycin (elfamycin antibiotic), *E. coli parE* conferring resistance to fluoroquinolones (fluoroquinolones (fluoroquinolone antibiotics), *flor* (phenicol antibiotics) and *dfrA1* (diaminopyrimidine antibiotic). The presence of these AMR genes confirms the isolates belongs to multidrug resistant category. Restriction-modification finder 1.1 shown the presence of the type 1 restriction enzymes targeting M.*Vch*21761 gene, recognition sequence (AAGNNNNNCATC) for methytransferases and S.*Vch*21761, recognition sequence (AAGNNNNNCATC) for specific subunit in type 1 restriction enzymes of *V. cholerae* strain 2012EL-2176 (Accession No: CP007634.1). Relevant genes and gene descriptions were given in supplementary table 2.

**Figure 1:**
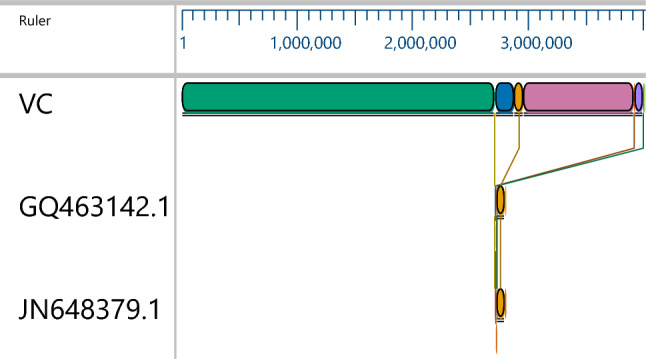
Multiple mauve alignment of ICEs of isolate (VC6) with the reference ICEs. From top to bottom: VC-isolate genome (VC6); GQ463142.1-*V. cholerae* Ind5 integrating conjugative element ICE*Vch*ind5 (Accession No: GQ463142.1); JN648379.1- *Vibrio cholerae* strain VC1786ICE (Accession No: JN648379.1). The putative ICE elements of reference ICEs shown as homologous block and are linked to the ICE of isolate genome connected by line. Image is generated by MegAlign Pro sequence alignment software.

**Figure 2:**
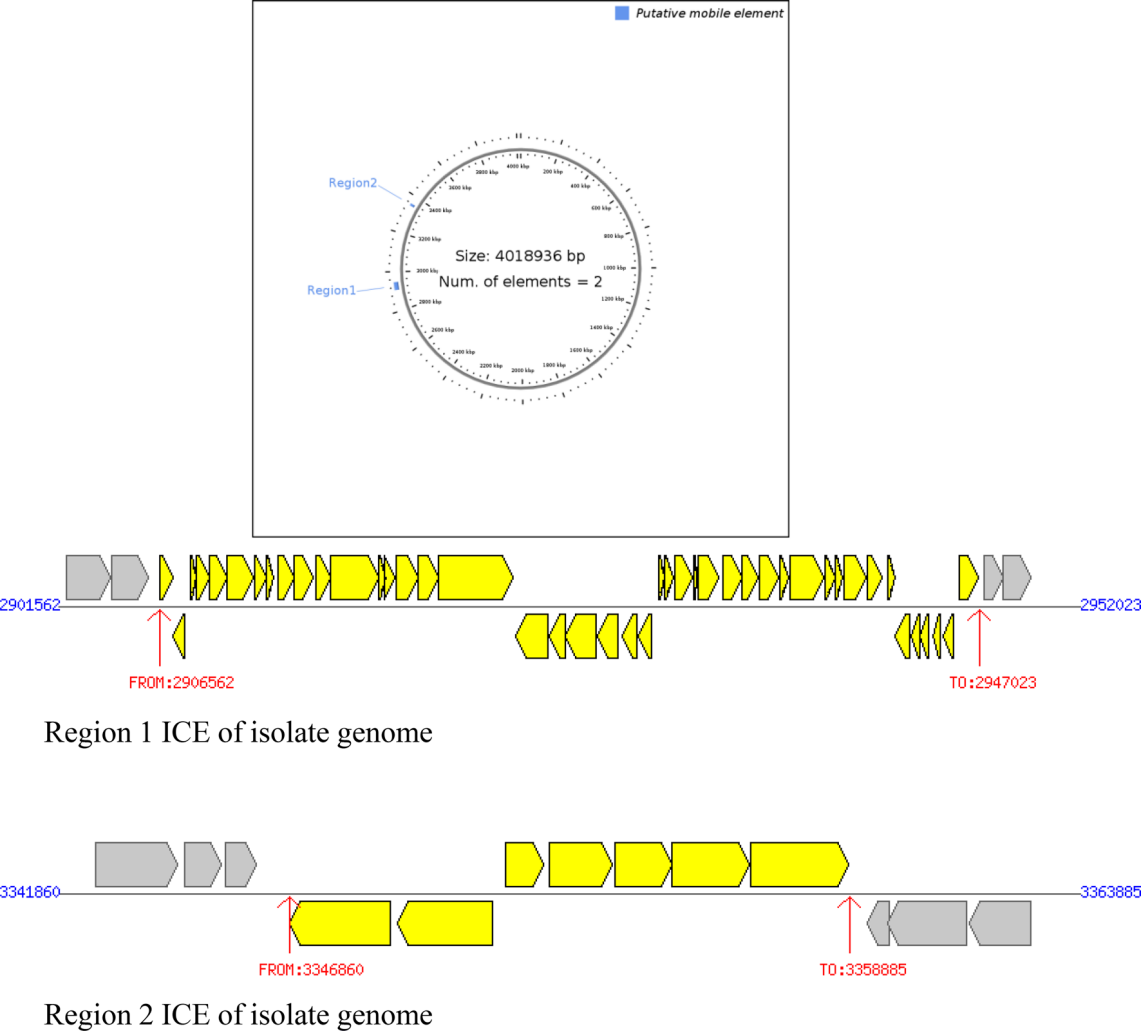
(**A**) Circular genome view of the isolate VC6 depicting the ICE region. **B** ICE element of the VC6 genome. **A**, (**B**): ICE element of the isolate genome found in between 2906562 to 2947023 bp and 3346860 bp to 3358885 bp with a total length of 40,462 bp in region 1 and 12026 bp in region 2. Region 1 and 2 of the ICE elements contained 44 and 7 genes constituting a total of 51 genes predicting various phenotypes of genes found in the SXT/R391 Family of ICEs.

Mobile genetic elements detected in the isolate were ISVsa3, ISVch1, ISVch8 and ISVch5. IS Vsa3 carries the resistance genes *sul2, floR, str1 and Str2* (aph (3’)-Ib, aph(6)-Id) (Table 4). ICEs found in the genome of the isolate are given in supplementary table 3 with its predictive phenotypes with identical genome ICE*Vch*Hai1 of the *V. cholerae* strain VC1786ICE (JN648379.1). The genetic relatedness of the ICEs showed high homology to ICE*Vch*Hai (*V. cholerae* strain VC1786 ICE genomic sequence JN648379.1), ICE*Vch*Ind5 (*V. cholerae* Ind5 integrating conjugative element ICE*Vch*Ind5, complete sequence (GQ463142.1) by multiple mauve alignment (Fig. 1). ICEfinder predicted 88 and 67 proteins in *V. cholerae* strain VC1786ICE genomic sequence (JN648379.1) and *V. cholerae* Ind5 integrating conjugative element ICE*Vch*ind5, complete sequence (GQ463142.1) respectively. Among these 44 and 7 proteins were found in region 1 and 2 of ICEs of isolate genome respectively constituting a total of 51 proteins (Fig. 2A,B) in the isolate genome.

### MLST typing

Core genome cgMLST and MLST analysis revealed that the isolate belong to ST 69, the pandemic sequence type commonly found in the clinical outbreak with *adk*, *gyrB*, *mdh,metE, pntA, purM, pyrC* genes with 100% identity and coverage with the alleles of 7,11,4, 37,12,1, and 20 respectively. MLST profiling of the isolate is given in Table 3.

**Table 3:** MLST profiling of *V. cholerae* isolate VC6 showing sequence type (ST) 69.

Locus	Identity	Coverage	Alignment length	Allele length	gaps	Allele
*adk*	100	100	416	416	0	7
*gyrB*	100	100	431	431	0	11
*mdh*	100	100	421	421	0	4
*metE*	100	100	591	591	0	37
*pntA*	100	100	431	431	0	12
*purM*	100	100	476	476	0	1
*pyrC*	100	100	449	449	0	20
*pntA*	100	100	431	431	0	12
*purM*	100	100	476	476	0	1
*pyrC*	100	100	449	449	0	20

### Comparison with reference genomes

**Figure 3:**
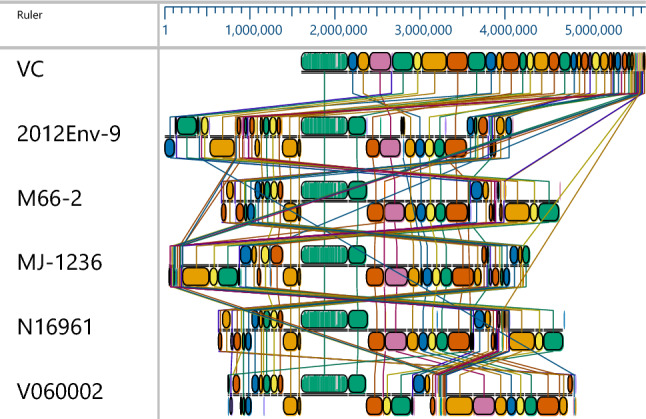
Multiple mauve genome alignment view of the isolate VC6 and five other reference genomes using progressive mauve algorithm using MegAlign Pro sequence alignment software. From top to bottom: VC- Isolate genome (VC6); 2012Env-9- *V. cholerae* strain 2012Env-9; M66-2- *V. cholerae* strain M66-2; MJ-1236- *V. cholerae* strain MJ-1236; V060002- *V. cholerae* strain V060002 strain. The genomic sequences were arranged into locally collinear blocks with homologous region are connected by lines. Homologous genomic blocks including inverted regions are differently positioned in each genome.

**Figure 4:**
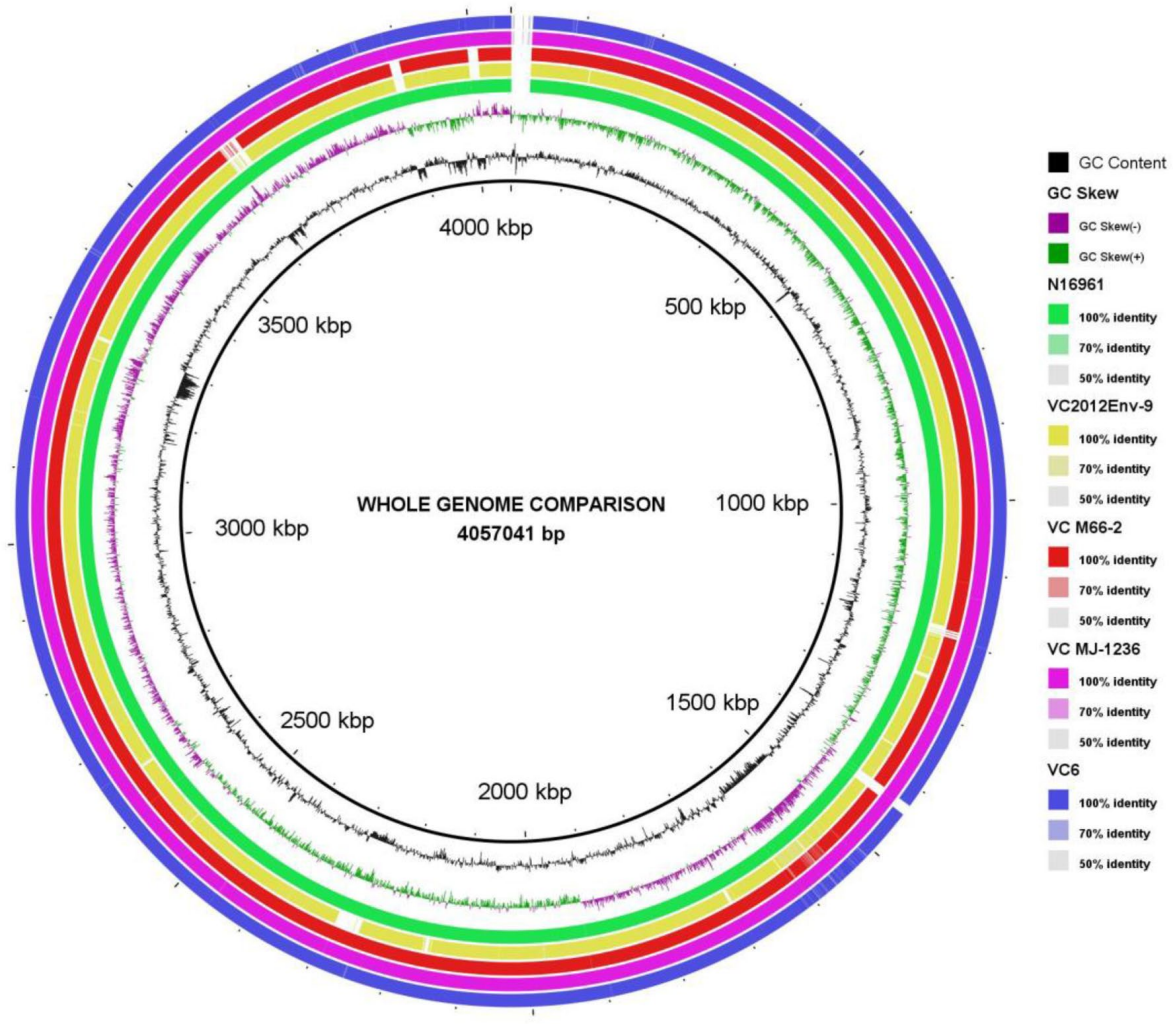
BLAST comparison of isolate genome with reference genomes using Blast Ring Image Generator (BRIG). *Vibrio cholerae* strain V060002 strain used as reference genome. From inside to outside Ring 1: GC content; Ring 2: GC Skew; Ring 3: BLAST comparison with *V. cholerae* O1 biovar El Tor strain N16961; Ring 4: BLAST comparison with *V. cholerae* strain 2012Env-9; Ring 5: BLAST comparison with *V. cholerae* strain M66-2; Ring 6: BLAST comparison with *V. cholerae* strain MJ-1236; Ring 7: BLAST comparison with Isolate genome (VC6).

The whole genome of the isolate was compared with reference genome to understand the evolution and relationship to pre 7th pandemic clone (*V. cholerae* M66-2 complete sequence NC_12578.1/NC-012580.1), seventh pandemic clone and it variant (*V. cholerae* O1 biovar El Tor strain N16961 complete sequence AE003852.1/AE003853.1; *V. cholerae* strain V060002 chromosome, complete genome NZ_AP018677.1; *V. cholerae* MJ-1236 complete sequence NC_012668.1/CP001486.1). Due to the absence of cholera prophage in the genome, we also included the nontoxigenic O1 strain of environmental origin (*V. cholerae* strain 2012Env-9 complete sequence CP012997.1/CP012998.1). The mauve genome alignment divided the genome in to regions of high homology with reference genomes (Fig. 3). The relationship of the core genome of the isolate with reference genomes were assessed and circular view of the genome comparison with similarity was presented in Fig. 4. Brig analysis showed the isolate genome shares maximum similarity with the reference genomes. However, significant differences were also noted in terms of virulomes, resistomes and genomic islands. Both pandemic islands, VSP1 and VSP2 were found in the isolate genome, N16961, MJ1236 and V060002 strains where as it was absent in the 2012Env-9 strain, and M66-2 strain. Similarly, well-defined *ctx* prophage unit were present in N16961, MJ-1236 and V060002 genome where as its absence was noted in the isolate genome, 2012Env-9 strain, and M66-2 strain. The resistome analysis revealed that the isolate genome possesses multidrug resistance genes similar to resistome profile of MJ-1236 strain. The detailed distribution of various genes was given in Table 4.

**Table 4:** List of various genes found in isolate and reference genomes.

Isolate name/accession No	Source	Species specific gene	Serogroup specific gene	Biotype specific genes	7th pandemic gene	Virulence genes	Pathogenicity islands	Antibiotic resistance genes
NC_012667.1 /NC_012668.1 *Vibrio cholerae* MJ-1236 complete sequence	Clinical	*ompW*	*rfb*-O1	*tcpA*_*et3,*_* ctxB-1, rstR*_*cc*_	VC2346	als,*mshA, toxR, ompT, rtxA, VgrG, ompU, ctxA,* *ace, zot,* *makA, VasX, hlyA*	VSP2_VC0498, VSP2_VC0493, VPI2_VC1809, VPI2_VC1776, VSP1_VC0180, VPI2_VC1783, VSP2_VC0514, VPI1_VC0819, VSP2_VC0512, VPI2_VC1790, VPI2_VC1758, VPI1_VC0840, VSP2_VC0516, VSP1_VC0178, VPI2_VC1765, VSP2_VC0502, VPI2_VC1760, VSP2_VC0490, VPI1_VC0827, VSP1_VC0175, VPI1_VC0847, VSP1_VC0183, VSP1_VC0185, VSP2_VC0504, VSP1_VC0185, VPI2_VC1790, VSP1_VC0183, VSP1_VC0180, VSP1_VC0175, VSP1_VC0178	*catB9* VC1786ICE9_*floR,* *dfrA1,* *sul2,* *parC,* *gyrA,* *strB,* *parE,* *strA*
VC6	This study	*ompW*	*rfb*-O1	*tcpA*_*et3*_	VC2346	*mshA, ompT, rtxA, als,* *makA, VasX, ompU, hlyA, VgrG, toxR*	VSP1-VC0180, VSP2-VC0516, VSP2-VC0514, VSP2-VC0512, VPI2-VC1760, VSP1-VC0185, VSP2-VC0502, VPI2-VC1790, VPI-VC0827, VPI1-VC0847, VSP1-VC0178, VPI2-VC1776, VSP1-VC0183, VSP2-VC0498, VPI2-VC1765, VPI1-VC0819, VSP2-VC0504, VPI2-VC1758, VPI2-VC1809, VPI1-VC0840, VSP2-VC0493, VSP1-VC0175, VSP2- VC0490, VPI2-VC1783	VC1786ICE9-*floR,* *strB,* *strA,* *parE,* *dfrA1,* *gyrA,* *parC,* *sul2,* *catB9*
NZ-AP018677.1 *V. cholerae* strain V060002 chromosome, complete genome	Clinical	*ompW*	*rfb*-O1	*ctxB-1, rstR*_*cc*_*, tcpA*_*et3*_	VC2346	*mpU, VgrG, VasX, ctxA,* *zot, makA, mshA, toxR, ompT, hlyA,* *rtxA, als,* *ace*	VSP1-VC0185, VSP2-VC0514, VSP1-VC0180, VSP2-VC0490, VSP2-VC0498, VSP2-VC0493, VPI2-VC1790, VSP2-VC0502, VPI1-VC0840, VSP2-VC0504, VSP2-VC0512, VSP1-VC0178, VPI2-VC1765, VSP1-VC0175, VPI2-VC1776, VPI2-VC1783, VSP1-VC0183, VPI2-VC1809, VPI1-VC0819, VPI1-VC0847, VPI2-VC1760, VPI2-VC1758, VSP2-VC0516, VPI1-VC0827	*gyrA,* *catB9,* *parC,* *parE*
AE003852.1/ AE003853.1 *.V.* *cholerae* O1 biovar El Tor strain N16961, complete sequence	Clinical	*ompW*	*rfb*-O1	*rstR*_*et*_*, tcpA*_*et3*_*, ctxB-3*	VC2346	*ctxA, mshA,* *als, VgrG, toxR, ompT, ompU, rtxA, zot, ace, makA, VasX,* *hlyA*	VSP2-VC0516, VPI2-VC1758, VSP1-VC0185, VPI2-VC1809, VSP2-VC0502, VSP1-VC0183, VPI2-VC1776, VSP2-VC0514, VSP2-VC0498, VPI2-VC1783, VSP1-VC0180, VSP2-VC0493, VPI1-VC0847, VPI1-VC0819, VPI1-VC0840, VPI1-VC0827, VSP2-VC0512, VPI2-VC1760, VSP2-VC0504, VSP1-VC0175, VSP1-VC0178, VPI2-VC1765, VSP2_VC0490, VPI2-VC1790, VPI2_VC1790	*parE,* *gyrA,* *parC* *catB9*
NC-012578.1/NC- 012,580.1 *V. cholerae* M66-2 complete sequence		*ompW*	*rfb*-O1	*tcpA-et3*	none	*als,* *ompU, VgrG, ompT, toxR, mshA, rtxA,* *hlyA, VasX, makA*	VPI2-VC1760, VPI2-VC1790, VPI2-VC1758, VPI2-VC1776, VPI1-VC0819, VPI2-VC1783, VPI2-VC1765, VPI1-VC0847, VPI2-VC1809, VPI-VC0840, VPI1-VC0827, VPI2-VC1790	*parC,* *parE,* *gyrA*
CP012998.1 / CP012997.1 *V.* *cholerae* strain 2012Env-9 complete sequence	Environmental	*ompW*	*rfb*-O1	none	none	*ompU,* *rtxA,* *als,* *mshA,* *toxR,* *ompT,* *hlyA*	VPI1-VC0819, VPI1-VC0847, VPI2-VC1758, VPI2-VC1790, VPI2-VC1760, VPI2_VC1790	*gyrA,* *parC,* *parE,* *catB9*

### Phylogenetic analysis

**Figure 5:**
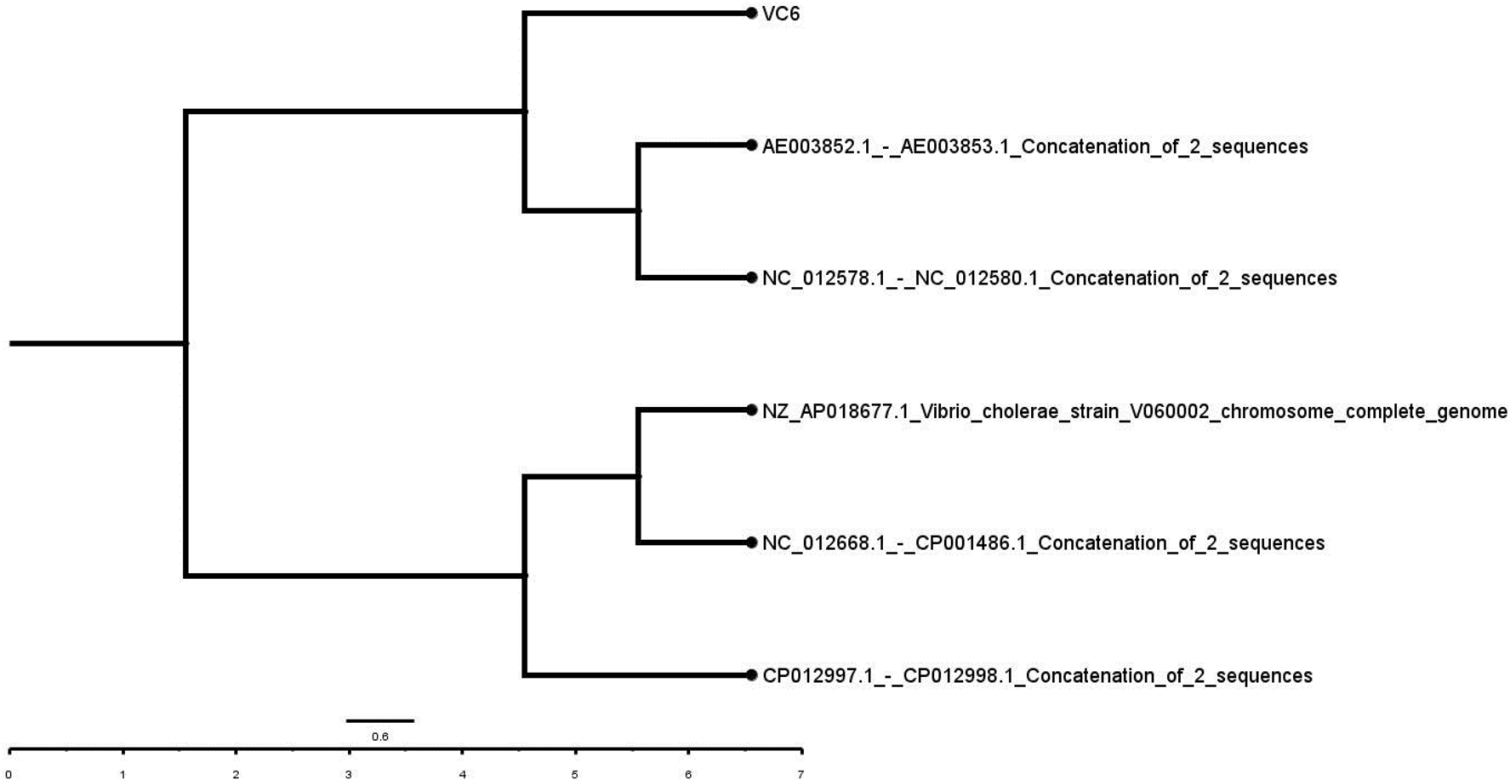
Bayesian phylogenetic tree of the isolate genome (VC6) with reference genomes. Unrooted bayesian phylogenetic tree with strict clock model was used for assessing the relationship among the isolate VC6 and the representative reference strains. The concatenated sequences of the both the chromosomes of the isolate were used along with full genome and/or concatenated sequences of reference genomes available in NCBI database (*V. cholerae* M66-2 complete sequence NC_012578.1/NC-012580.1; *V. cholerae* O1 biovar El Tor strain N16961 complete sequence AE003852.1/AE003853.1; *V. cholerae* strain V060002 chromosome, complete genome NZ_AP018677.1; *V. cholerae* MJ-1236 complete sequence NC_012668.1/CP001486.1; and *V. cholerae* strain 2012Env-9 complete sequence CP012997.1/CP012998.1). Posterior probability values of ≥ 0.91 are indicated as dark nodes Bayesian analysis shows the possible lineage of VC6 genome to the nearest clade containing seventh pandemic O1 El Tor biovar N16961 strain and pre-pandemic M66-2 strain.

**Figure 6:**
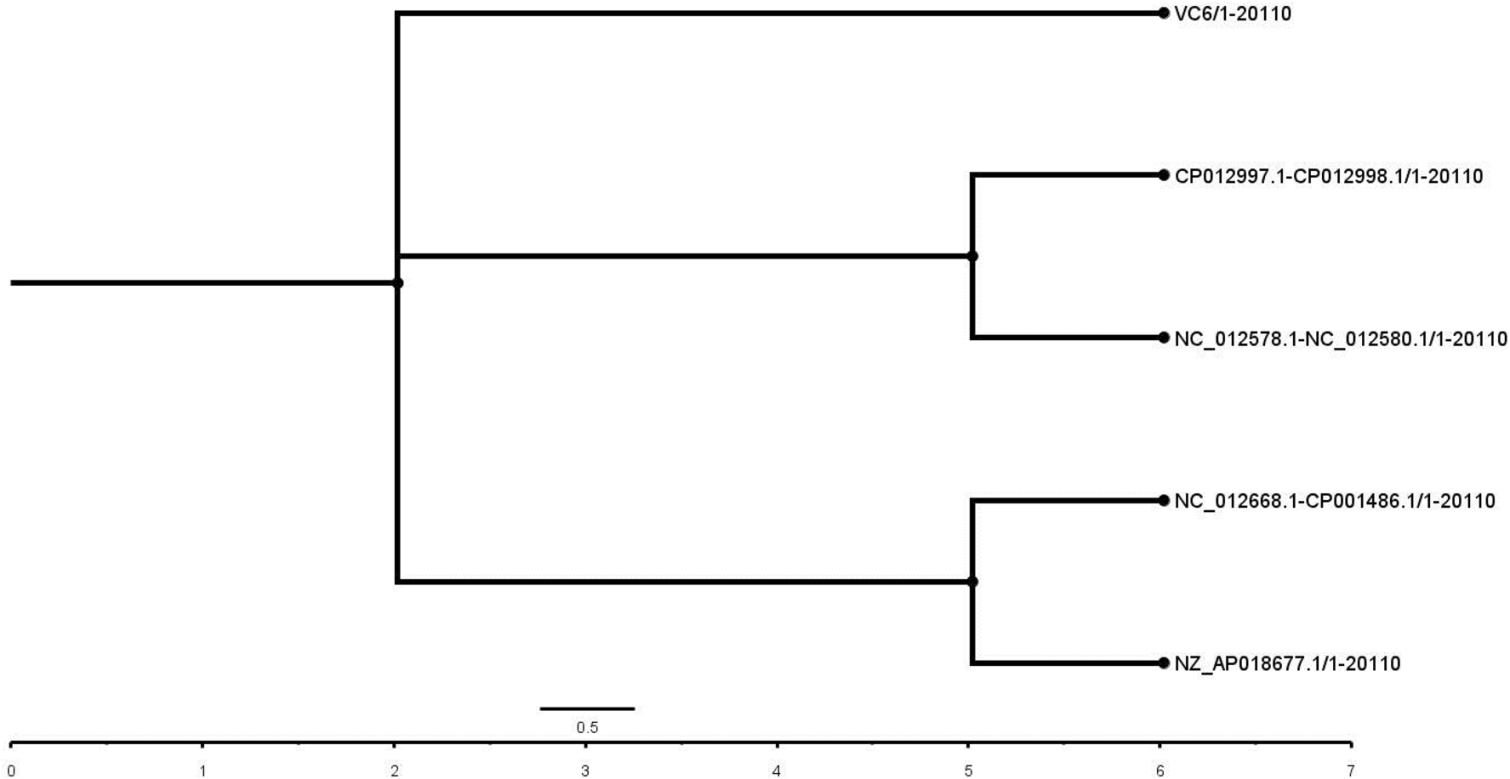
SNP based phylogenetic tree obtained from CSIPhylogeny-1.4. The concatenated sequences of both the chromosomes of *Vibrio cholerae* O1 biovar El Tor strain N16961 (AE003852.1/AE003853.1) was used as reference genome. The tree is visualized by using Fig.Tree v1.3. Default input parameters such as minimum Z score of 1.96, read mapping quality of 25, and SNP quality of 30 were used for the construction of SNP tree. Total number of positions and percentage similarity of the investigated genomes with reference genome in SNP tree is given in Table 5.

**Figure 7:**
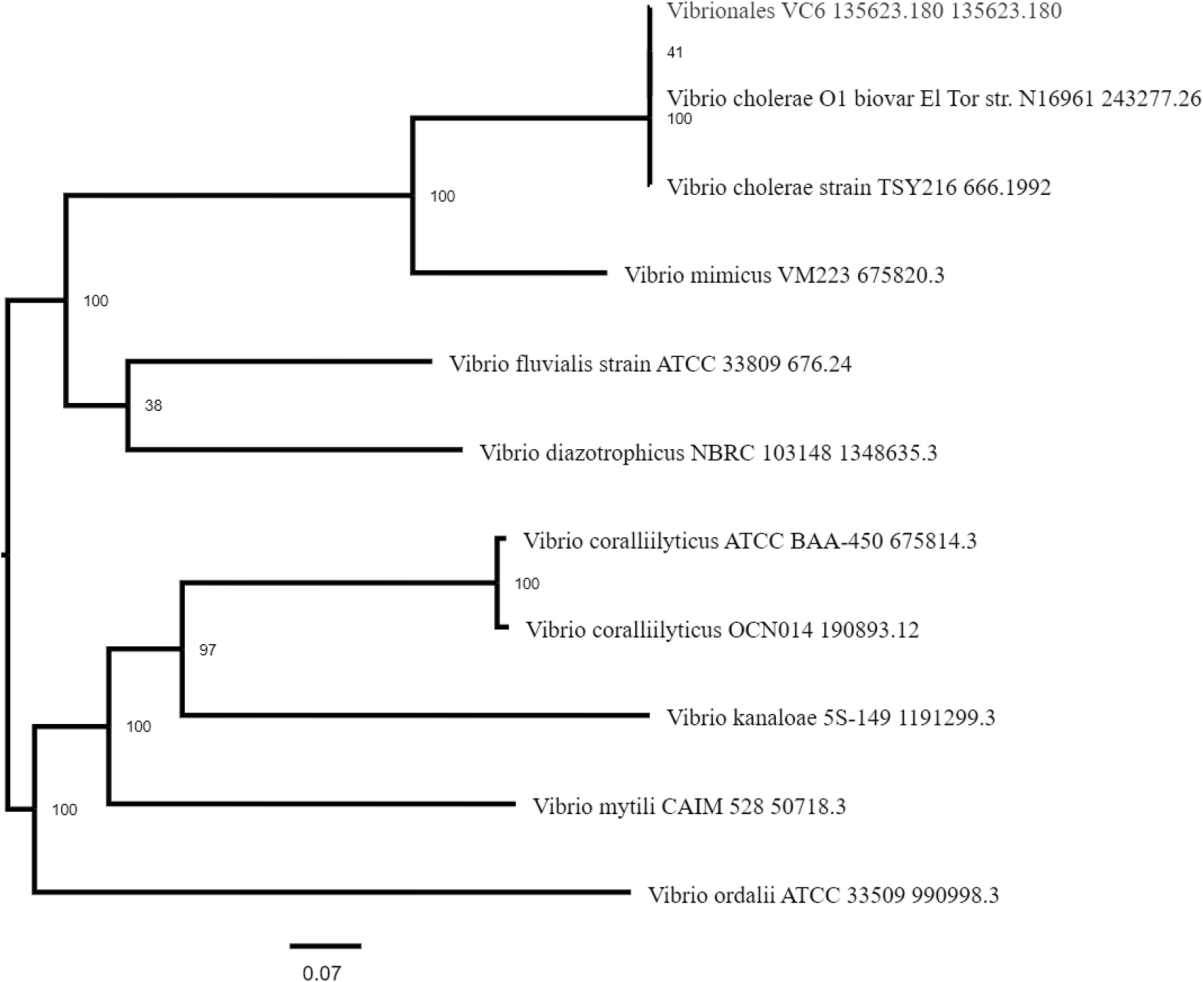
Phylogenetic tree derived as a part of PATRIC comprehensive genome analysis report of the isolate genome. The tree is generated based on global protein families in which PATRIC PGFams automatically selects reference and representative genomes based on k-mer based functional assignments followed by Markov cluster algorithm. Bootstrap values were calculated by standard bootstrapping method and nodes are labelled with bootstrap values.

Bayesian phylogenetic analysis revealed the most probable pandemic lineage of the isolate and its relationship with other reference genomes (Fig. 5). Similarly, SNP based phylogenetic analysis of the isolate genome with five other closely related genomes were analysed in CSI Phylogeny Version 1.4 tool with strain *V. cholerae* O1 El Tor N16961 strain as reference genome (Fig. 6). A total of 92.4% of the reference genome were covered in all the investigated genome with a total of 3,727,036 positions. Percentage similarity of the isolate genome with the reference genome was 98.77% whereas with nontoxigenic O1 strain of environmental origin (*Vibrio cholerae* strain 2012Env-9) was only 94.68%. Number of positions that are shared between each investigated genome and the reference genome is given in Table 5. Phylogenetic analysis based on PATRIC global protein families showed the isolate genome similarity with *V. cholerae* O1 El Tor biovar N16961 strain (Fig. 7).

**Table 5:** Total number of positions and percentage similarity of the investigated genomes with reference genome in SNP tree.

File	Valid positions	Percentage similarity
*Vibrio cholerae* MJ-1236 complete sequence NC_012668.1/CP001486.1	4,009,762	99.41
*V. cholerae* strain 2012Env-9 complete sequence CP012997.1/CP012998.1)	3,819,080	94.68
*V. cholerae* strain V060002 chromosome, complete genome NZ_AP018677.1	4,050,964	100
VC6	3,984,098	98.77
*V. cholerae* M66-2 complete sequence NC_012578.1/NC- 012,580.1	3,943,936	97.78

## Discussion

Aquatic environment plays a significant role in the evolution, transmission of *V. cholerae.* Several reports are available for the diverse antigenic, toxigenic, as well as phenotypically variant/hybrid *V. cholerae* for spreading the cholera across the globe^15–17^. The *V. choleare* isolate VC6 was recovered from coastal water near to Thoppumpady fishing harbor, Cochin, Kerala, India. This harbor may be considered as one of the strategic location for the isolation of clinically relevant pathogen due to its close proximity to primary care hospitals and municipal waste waters thereby heightened the risk of hospital effluents reaching to the nearby water bodies. Previous research has provided substantial evidence of highlighting the role of hospital wastewater in the propagation of clinically relevant antimicrobial resistant pathogens^54^. However, as of now there has been no report available on the occurrence of clinical outbreak of cholera from this location. Thoppumpady harbor serves as a key hub for both wholesale and retail fish trade, encompassing river-caught and marine fish varieties. Interestingly, the seafood samples screened from the same source were devoid of virulence characteristics such as biotype specific and pathogen specific genes. The physical observation of seafood samples at the fishing harbour during sample collection did not show any sign of onset of bacterial infection. However, the unpredictable nature of anthropogenic activities in the harbor, source tracking the origin of VC6 posed a significant challenge.

Whole genome sequence analysis of the isolate VC6 resulted in the absence of cholera prophage region; both core and RS2 region. Core region encodes biotype specific *ace*, *zot*, *ctxA*/*ctxB* genes which required for the morphogenesis of *ctx* phage particles and RS2 element contain *rstR*, *rstA* and *rstB* toxin genes which required for the replication and regulatory functions of the *ctx* phage^18^. However, the isolate harboured *rfb-*O1, *tcpA* (El Tor), cytotoxic *rtxA* (repeat in toxin) gene. Seventh pandemic strains of wave 1, wave 2, and wave 3 V*. cholerae* harbours both core and RS2 region of the cholera prophage, as well as the *tcpA*, and *rtx* gene^12^. The wave is described by the presence of specific *ctx-1, ctx-2* and *ctx-3* type phage regions in wave 1, 2 and 3 respectively, in which the major differences are found in the genomic sequences of *rstA* and *ctxB* genes^8^. Since 2006, several O1 EI Tor variants have been emerged such as *ctx*-3b, *ctx-*4, *ctx*-5 and *ctx*-6, and *ctx*-6b for severe diarrhoea in humans^19^. The wave of the isolate could not be determined in the present study due to the absence of the cholera prophage region. The other molecular characteristics of the seventh pandemic strains were present in the isolate genome such as pandemic specific VC2346 gene, pathogenicity islands (VPI1 and VPI2), and pandemic island (VSP1 and VSP2). The presence of this pathogen in aquatic environment is quite surprising. Interestingly, *ctx* negative O1 known phyletic lineage emerging from the pre-pandemic ancestor of the PG clades, was recovered from Haitin aquatic environment^38^. Bhandari et al.^13,26^ reported that Queensland waterways harboured highly diverged O1 and Non-O1 *V. cholerae* including *ctx* positive toxigenic, non-O1 strains with pandemic characteristics. In an epidemiological investigation of cholera cases in Hangzhou (China) revealed the presence of *ctx* negative, *tcpA* positive isolates with pandemic characteristics in clinical cases with mild to moderate symptoms. However, the *ctx* negative and *tcpA* positive isolates recovered form environmental sources were reported only for the presence of VPI2 genetic elements, and the pandemic features were not detected^25^. In a cholera outbreak study conducted in Kottayam district of Kerala in 2001, reported two environmental O1 strains lacking both *ctx* and *tcpA* genes and clinical O1 toxigenic strains possessing *ctx* and *tcpA* genes. Based on the typing techniques, they concluded that the environmental non toxigenic O1 strains might be ancestors of the clinical toxigenic O1 strain^14^. Anandan et al.^44^ reported that clonal expansion of clinical strains of *V. cholerae* O1 seventh pandemic obtained from the past 20 years in India has resulted in ST69 strains along with ST69 *V. cholerae* O139.

This is the first report globally for the environmental *ctx* negative O1 El Tor strain having pandemic characteristics and belongs to the pandemic sequence type (ST) 69. In addition to cholera prophage unit, the isolates also lack other accessory toxin genes of pandemic O1 El Tor strains such as *zot, ace, rstR* genes. The specific marker of the seventh pandemic clone in the genome shows 100% identity with pandemic gene VC2346 of the *V. cholerae* O1 biovar El Tor strain N16961 chromosome I, complete sequence (Accession No: AE003852.1). The role of SXT/R391 family related ICEs in recent pandemic variants of *V. cholerae* has been well studied^32^. The genomic analysis of the isolate genome showed the presence of the ICE element in their genome. The isolate possesses the *prfC* gene which shows the presence integration site of the SXT element^27^.

The acquisition of VSP1 and VSPII and *ctx*_et_ genes in the genome of *V. cholerae* is the requirement of the isolate to be seventh pandemic category^20^. Dziejman et al.^50^ first described VSP1 and VSP2 in seventh pandemic *V. cholerae* isolates and later the acquisition of these islands is explained by the phenomenon of lateral gene transfer event^51,52^. It was reported that the GC content of the classical and El Tor strains is 40%. However, the presence of VSP1 and VSP2 gene clusters in pandemic isolates shows high GC content of 47%^21^. The genome analysis of the isolate VC6 also reveals the high GC content of 47%. Initially VSP2 identified as gene cluster of genes from VC0490 to VC0497^50^ constituting 7.5 kb size and later by O'Shea et al.^21^ found additional genes to VC0516 where the site of integration lies and contributing a total size of VSP2 gene of about 26.9 kb. The isolate VC6 possess VC0516 and predicted the gene function integrase, however, the other important gene VC0503 which predict the phenotype peptidoglycan endopeptidase is absent. VSP2 variant with epidemic potential have also been reported in many parts of the world such as Haiti, South Asia countries, Western Africa and South America^33^. The genes in VSP1 islands of N16961 genome were also found in the isolate (VC6) genome. The absence of VSPI in clinical isolates have been reported and none of the environmental isolates possesses the VSP1 in their genome^47^.

Genome analysis of *V. cholerae* isolate reveals characteristics of pandemic features. However, the pathogenicity of this variant need to be investigated. The absence of toxigenic genes in the emerging pandemic variants in the aquatic environment needs the exploitation of pathogenic significance. In addition to these, the MLST analysis revealed that isolate sequence type 69 which is the common sequence type of pandemic *V. cholerae* strain found in India. The sequence type 70 is commonly found in nontoxigenic strains and are closely related to ST 69 with difference only in the allele of *pyrC* gene^13,26^. The presence of this pathogen in the aquatic environment poses great concern of public health and demanded detailed genomic analysis and epidemiological surveillance. The El Tor strains have good adaptive ability and survivability to the environment compared to classical strains even though the more severe form of cholera is credited in comparison to classical strains^28^. The environmental persistence of the El Tor strain is achieved through drug resistance, horizontal acquisition of pandemic islands in which VSP1 is directly linked to environmental fitness of the pathogen to the host^29,30^. Out of 30 ORFs of VSP2, two genes such VC0516 (integrase) and VC0510 (peptidoglycan endopeptidase) have validated functions and the isolate VC6 possess the VC0516 along with uncharacterized genes of predicted phenotypes transcriptional regulators, ribonuclease H, type1V pilin, DNA repair protein, and methyl accepting chemotaxis proteins. It was reported that, the genes of VSP2 are not expressed in laboratory condition and yet it induced only in environmental conditions triggering some unknown signal^31^. Recent studies shown that the presence of mobile genetic elements possesses antiphage elements which encodes phage defense genes. LeGault et al.^11^ reported that SXT elements in *V. cholerae* harbours anti phage genes in additions to antimicrobial resistance elements. Similarly, O’Hara et al.^53^ reported that the first gene of VSP1 VCO175 encoding AVcD (deoxycytidine deaminase) also has the potential of inhibiting phage attack. The *rfb*-O1 gene and *tcpA* gene of the isolate shows 100% identity to *V. cholerae* strain N16961 suggesting a close relation in evolutionary point of view.

## Conclusion

The genome analysis reveals the dynamic change and emergence of new strain of seventh pandemic *V. choleare* O1 El Tor via the absence of *ctx* prophage region, acquisition of mobile genetic elements and AMR genes. The findings in this study revealed that coastal water contributes the evolution of new variants of *V. cholerae.* The emergence of several variants of *V. cholerae* in recent years becomes a huge threat and may lead to the possibility of eighth cholera pandemics. The pathogenic potential of such strains needs to be continuously monitored on real time basis considering its pandemic spread. The detailed study on genomic features and toxicity aspects of this strain will be undertaken in future research.

### Supplementary Information

Supplementary Information.

## Data Availability

The raw sequences of the isolate genome was deposited to NCBI SRA database under bio project id: PRJNA970544 with bio sample id: SAMN35005786. Web link to access the data: https://www.ncbi.nlm.nih.gov/bioproject/PRJNA970544
