# Supplementary material for: Emergence of multidrug resistant, ctx negative seventh pandemic Vibrio cholerae O1 El Tor sequence type (ST) 69 in coastal water of Kerala, India
Source: Sci Rep. 2024 Jan 23;14:2031. doi: 10.1038/s41598-023-50536-z (PMC10805778; doi:10.1038/s41598-023-50536-z)
Supplement: Supplementary file 1 — Supplementary Information. [file 41598_2023_50536_MOESM1_ESM.pdf]

Title: Emergence of multidrug resistant, *ctx* negative seventh pandemic *Vibrio cholerae* O1 El Tor sequence type (ST) 69 in coastal water of Kerala, India

Ahthors:

Minimol V. Ayyappan, Pankaj Kishore, Satyen Kumar Panda, Anuj Kumar, Devananda Uchoi, Ranjit Kumar Nadella, Himanshu Priyadarshi, Mohan Chitradurga Obaiah, Dybin George, Muneeb K. Hamza, Sreelekshmi K. Ramannathan, and Ravishankar C N

Supplementary Table 1: Sequence information of the isolate VC6

S. No	Sequence Information	VC6
**Genome Statistics**		
1.	Contigs	75
2.	Genome Length	4018937
3.	GC Content	47.4938
4.	Contig L50	6
5.	Contig N50	204396
**Annotation Statistics**		
1.	tRNA	75
2.	rRNA	4
3.	CDS	3724
4.	CDS Ratio	0.92661315
5.	Hypothetical CDS	633
6.	Hypothetical CDS Ratio	0.2714823
7.	PLFAM CDS	3640
8.	PLFAM CDS Ratio	0.97744364
**Genome Quality**		
1.	Coarse Consistency	99.8
2.	Fine Consistency	99.4
3.	CheckM Completeness	100
4.	Genome Quality	Good

Supplementary Table 2: List of genes found in *Vibrio cholerae* isolate genome (VC6) with gene description

Genes found	
**Relevance**	**Gene(s)**
**Specie specific gene**	*ompW*
**Serogroup specific**	*rfbV*_O1
**Biotypespecific**	*tcpA*_et3
**7th Pandemic**	VC2346
**Virulence**	*als, VgrG, makA, rtxA, ompT, ompU, hlyA, toxR, VasX, mshA*
**Mobile Genetic Elements**	None
**Phage susceptibility**	None
**Pathogenicity Islands**	VPI1_VC0827, VSP1_VC0183, VPI2_VC1783, VPI2_VC1776, VPI2_VC1758, VPI2_VC1790, VSP1_VC0175, VSP1_VC0180, VPI2_VC1765, VSP2_VC0493, VSP2_VC0514, VSP1_VC0178, VPI1_VC0847, VPI1_VC0819,VSP2_VC0490, VSP2_VC0504, VPI2_VC1809, VPI2_VC1760, VSP2_VC0512, VSP2_VC0502, VSP2_VC0498,VSP2_VC0516, VSP1_VC0185, VPI1_VC0840
**Antibiotic Resistance**	*sul2, parE, dfrA1, strA, catB9, strB, parC, gyrA, VC1786ICE9_floR*

Genes description						
**Gene for 7th Pandemic**						
***Vibrio cholerae* Gene**	**Identity**	**Query /Template length**	**Contig**	**Position in contig**	**Predicted phenotype**	**Accession number**
VC2346	100	687 / 687	VC_6_contig_15	46064..46750	7th pandemic *V. cholerae* strain specific gene (vc2346)	AE003852

**Pathogenicity Islands**						
***Vibrio cholerae* Gene**	**Identity**	**Query /Template length**	**Contig**	**Position in contig**	**Predicted phenotype**	**Accession number**
VPI1_VC0819	100	1626 /1626	VC_6_contig_10	58957..60582	Aldehyde dehydrogenase (*Vibrio cholerae* pathogenicity island 1, VPI-1)	AE003852
VPI1_VC0827	100	411 / 411	VC_6_contig_10	45948..46358	Toxin co-regulated pilus biosynthesis protein H (*Vibrio cholerae* pathogenicity island 1, VPI-1)	AE003852
VPI1_VC0840	100	1881 /1881	VC_6_contig_10	32479..34359	Accessory colonization factor *AcfB* (*Vibrio cholerae* pathogenicity island 1, VPI-1)	AE003852
VPI1_VC0847	100	1269 /1269	VC_6_contig_10	21729..22997	Integrase, phage family (*Vibrio cholerae* pathogenicity island 1, VPI-1)	AE003852
VPI2_VC1758	100	1236 /1236	VC_6_contig_8	39399..40634	Integrase, phage family (*Vibrio cholerae* pathogenicity island 2, VPI-2)	AE003852
VPI2_VC1760	100	2823 /2823	VC_6_contig_8	35895..38717	Helicase, putative (*Vibrio cholerae* pathogenicity island 2, VPI-2)	AE003852
VPI2_VC1765	100	3069 /3069	VC_6_contig_8	27869..30937	Type I restriction enzyme *HsdR*, putative (*Vibrio cholerae* pathogenicity island 2, VPI-2)	AE003852
VPI2_VC1776	100	897 /897	VC_6_contig_8	9635..10531	N-acetylneuraminate lyase, putative (*Vibrio cholerae* pathogenicity island 2, VPI-2)	AE003852

VPI2_VC1783	100	1137 /1137	VC_6_contig_8	3841..4977	N-acetylglucosamine-6-phosphate deacetylase (*Vibrio cholerae* pathogenicity island 2, VPI-2)	AE003852
VPI2_VC1790	100	306 /345	VC_6_contig_46	878..1183	Transposase *OrfAB*, subunit A (*Vibrio cholerae* pathogenicity island 2, VPI-2)	AE003852
VPI2_VC1809	100	231 /231	VC_6_contig_2	487797..488027	Transcriptional regulator, putative (*Vibrio cholerae* pathogenicity island 2, VPI-2)	AE003852
VSP1_VC0175	100	1599 /1599	VC_6_contig_14	101070..102668	Deoxycytidylate deaminase-related protein (*Vibrio cholerae* seventh pandemic island 1, VSP-1)	AE003852
VSP1_VC0178	100	1068 /1068	VC_6_contig_14	97606..98673	Patatin-related protein (*Vibrio cholerae* seventh pandemic island1, VSP-1)	AE003852
VSP1_VC0180	99.94	1755 /1755	VC_6_contig_14	94525..96279	ThiF domain-containing protein (*Vibrio cholerae* seventh pandemic island 1, VSP-1)	AE003852
VSP1_VC0183	100	2112 /2112	VC_6_contig_14	91540..93651	Uncharacterized protein (*Vibrio cholerae* seventh pandemic island1, VSP-1)	AE003852
VSP1_VC0185	100	1215 /1215	VC_6_contig_14	88631..89845	Transposase, putative (*Vibrio cholerae* seventh pandemic island1, VSP-1)	AE003852
VSP2_VC0490	100	1962 /1962	VC_6_contig_13	116029..117990	Uncharacterized protein (*Vibrio cholerae* seventh pandemic island2, VSP-2)	AE003852
VSP2_VC0493	100	876 /876	VC_6_contig_13	119918..120793	Uncharacterized protein (*Vibrio cholerae* seventh pandemic island2, VSP-2)	AE003852
VSP2_VC0498	100	441 /441	VC_6_contig_27	1559..1999	Ribonuclease HI, putative (*Vibrio cholerae* seventh	AE003852

					pandemic island2, VSP-2)	
VSP2_VC0502	100	525 /525	VC_6_contig_27	5080..5604	Type IV pilin, putative (*Vibrio cholerae* seventh pandemic island2, VSP-2)	AE003852
VSP2_VC0504	100	228 /228	VC_6_contig_9	839..1066	Uncharacterized protein (*Vibrio cholerae* seventh pandemic island2, VSP-2)	AE003852
VSP2_VC0512	100	1590 /1590	VC_6_contig_9	5281..6870	Methyl-accepting chemotaxis protein (*Vibrio cholerae* seventh pandemic island 2, VSP-2)	AE003852
VSP2_VC0514	100	1881 /1881	VC_6_contig_9	9136..11016	Methyl-accepting chemotaxis protein (*Vibrio cholerae* seventh pandemic island 2, VSP-2)	AE003852
VSP2_VC0516	100	1242 /1242	VC_6_contig_9	12742..13983	Phage integrase (*Vibrio cholerae* seventh pandemic island 2, VSP-2)	AE003852

**Other ICE genes**						
***Vibrio cholerae* Gene**	**Identity**	**Query /Template length**	**Contig**	**Position in contig**	**Predicted phenotype**	**Accession number**
VC1786ICE1	100	1854 /1854	VC_6_contig_9	168872..170725	Conserved hypothetical protein	JN648379
VC1786ICE10	100	540 /540	VC_6_contig_24	2817..3356	Putative transposase	JN648379
VC1786ICE14	100	1309 /1353	VC_6_contig_43	142..1450	Transposase	JN648379
VC1786ICE15	100	1212 /1212	VC_6_contig_44	36..1247	Transposase Mutator family	JN648379
VC1786ICE16_mutL	100	503 /504	VC_6_contig_11	1..503	MutL protein	JN648379
VC1786ICE17	100	270 /270	VC_6_contig_11	656..925	Error-prone repair proteinUmuC	JN648379
VC1786ICE18	100	450 /450	VC_6_contig_11	933..1382	DNA polymerase V	JN648379

VC1786ICE19	100	906 /906	VC_6_contig_11	2022..2927	DNA polymerase III subunit epsilon	JN648379
VC1786ICE2	100	1242 /1242	VC_6_contig_9	170855..172096	Phage integrase family protein	JN648379
VC1786ICE20	100	300 /300	VC_6_contig_11	3015..3314	Conserved hypothetical plasmid-related protein	JN648379
VC1786ICE21	100	921 /921	VC_6_contig_11	3632..4552	Conserved hypothetical protein	JN648379
VC1786ICE22	100	801 /801	VC_6_contig_11	4570..5370	Conserved hypothetical protein	JN648379
VC1786ICE23	100	3690 /3690	VC_6_contig_11	5915..9604	Conserved hypothetical protein	JN648379
VC1786ICE24	100	3741 /3741	VC_6_contig_11	9609..13349	Conserved hypothetical protein	JN648379
VC1786ICE25_*pglZ*	100	2304 /2304	VC_6_contig_11	13362..15665	*PglZ* domain protein	JN648379
VC1786ICE26	100	2052 /2052	VC_6_contig_11	15704..17755	ATP-dependent protease	JN648379
VC1786ICE27	100	771 /771	VC_6_contig_11	17782..18552	Conserved hypothetical protein	JN648379
VC1786ICE28	100	816 /816	VC_6_contig_11	18587..19402	Conserved hypothetical protein	JN648379
VC1786ICE29_*traI*	100	2151 /2151	VC_6_contig_11	19524..21674	Conjugal transfer pilus assembly protein *TraI*	JN648379
VC1786ICE3	100	975 /975	VC_6_contig_9	172370..173344	Putative PRTRC system protein D	JN648379
VC1786ICE30_*traD*	100	1821 /1821	VC_6_contig_11	21723..23543	Conjugal transfer pilus assembly protein *TraD*	JN648379
VC1786ICE31	100	561 /561	VC_6_contig_11	23553..24113	Conserved hypothetical protein	JN648379
VC1786ICE32	100	636 /636	VC_6_contig_11	24100..24735	integrating conjugative element membrane protein	JN648379
VC1786ICE33	100	588 /588	VC_6_contig_11	24762..25349	Conserved hypothetical protein	JN648379
VC1786ICE34_*traL*	100	282 /282	VC_6_contig_11	25638..25919	Type IV conjugative transfer system protein *TraL*	JN648379

VC1786ICE35_*traE*	100	627 /627	VC_6_contig_11	25916..26542	Type IV conjugative transfer system protein *TraE*	JN648379
VC1786ICE36_*traK*	100	900 /900	VC_6_contig_11	26523..27422	Conjugal transfer pilus assembly protein TraK	JN648379
VC1786ICE37_*traB*	100	1290 /1290	VC_6_contig_11	27425..28714	TraB pilus assembly family protein	JN648379
VC1786ICE38_*traV*	100	651 /651	VC_6_contig_11	28711..29361	Type IV conjugative transfer system protein *TraV*	JN648379
VC1786ICE39_*traA*	100	387 /387	VC_6_contig_11	29358..29744	Conjugal transfer pilus assembly protein *TraA*	JN648379
VC1786ICE4	100	444 /444	VC_6_contig_9	173809..174252	Conserved hypothetical protein	JN648379
VC1786ICE40	100	834 /834	VC_6_contig_11	29923..30756	Conserved hypothetical protein	JN648379
VC1786ICE41	100	939 /939	VC_6_contig_11	30749..31687	Conserved hypothetical protein	JN648379
VC1786ICE42	100	693 /693	VC_6_contig_11	31819..32511	Putative thiol	JN648379
VC1786ICE43_*traC*	100	2400 /2400	VC_6_contig_11	32511..34910	Type-IV secretion system protein *TraC*	JN648379
VC1786ICE44	100	348 /348	VC_6_contig_11	34903..35250	Conserved hypothetical protein	JN648379
VC1786ICE45_*trbI*	100	513 /513	VC_6_contig_11	35234..35746	K12062 conjugal transfer pilin signal peptidase *TrbI*	
VC1786ICE46_*traW*	100	1125 /1125	VC_6_contig_11	35757..36881	Type-F conjugative transfer system protein *TraW*	JN648379
VC1786ICE47_*traU*	100	1029 /1029	VC_6_contig_11	36865..37893	Conjugative transfer pilus assembly protein *TraU*	JN648379
VC1786ICE48_*traN*	100	3693 /3693	VC_6_contig_11	37896..41588	type-F conjugative transfer system mating-pair stabilization protein *TraN*	JN648379
VC1786ICE49	100	1617 /1617	VC_6_contig_11	41679..43295	Putative SMC protein	JN648379

VC1786ICE5	100	1173 /1173	VC_6_contig_9	174263..175435	UV repair DNA polymerase	JN648379
VC1786ICE50	100	756 /756	VC_6_contig_11	43375..44130	*IstB* ATP binding domain-containing protein	JN648379
VC1786ICE51	100	1509 /1509	VC_6_contig_11	44120..45628	Integrase catalytic subunit *IstA*	JN648379
VC1786ICE52	100	1080 /1080	VC_6_contig_11	45727..46806	Putative SMC domain protein	JN648379
VC1786ICE53	100	684 /684	VC_6_contig_11	46974..47657	Endonuclease I	JN648379
VC1786ICE54	100	603 /603	VC_6_contig_11	47766..48368	Conserved hypothetical protein	JN648379
VC1786ICE55_*ssb*	100	420 /420	VC_6_contig_11	49078..49497	Single-strand binding protein *ssb*	JN648379
VC1786ICE56_*bet*	100	819 /819	VC_6_contig_11	49577..50395	Phage recombination protein *Bet*	JN648379
VC1786ICE57	100	1017 /1017	VC_6_contig_11	50681..51697	Putative phage-type endonuclease	JN648379
VC1786ICE58_*cobs*	100	960 /960	VC_6_contig_11	51907..52866	Cobaltochelatase, *CobS* subunit	JN648379
VC1786ICE59	100	768 /768	VC_6_contig_11	52866..53633	Conserved hypothetical protein	JN648379
VC1786ICE6	100	1309 /2979	VC_6_contig_43	142..1450	Transposase Tn3 family protein	JN648379
VC1786ICE60	100	954 /954	VC_6_contig_11	53732..54685	Conserved hypothetical plasmid-related protein	JN648379
VC1786ICE61	100	441 /441	VC_6_contig_11	54747..55187	Conserved hypothetical protein	JN648379
VC1786ICE62	100	1656 /1656	VC_6_contig_11	55257..56912	Conserved hypothetical protein	JN648379
VC1786ICE63	100	498 /498	VC_6_contig_11	56997..57494	DNA repair protein *RadC*	JN648379
VC1786ICE64	100	342 /342	VC_6_contig_11	57494..57835	Conserved hypothetical protein	JN648379
VC1786ICE65	100	1074 /1074	VC_6_contig_11	57926..58999	Putative zinc-binding domain of primase-helicase family	JN648379

VC1786ICE66	100	708 /708	VC_6_contig_11	59089..59796	Conserved hypothetical protein	JN648379
VC1786ICE67	100	702 /702	VC_6_contig_11	60469..61170	Conserved hypothetical protein	JN648379
VC1786ICE68	100	960 /960	VC_6_contig_11	63602..64561	Integron integrase	JN648379
VC1786ICE69_*traF*	100	945 /945	VC_6_contig_11	64818..65762	Type-F conjugative transfer system pilin assembly protein *TraF*	JN648379
VC1786ICE7	100	1494 /1494	VC_6_contig_24	6147..7640	Transposase *InsA*	JN648379
VC1786ICE70_*traH*	100	1389 /1389	VC_6_contig_11	65765..67153	Conjugative transfer pilus assembly protein *TraH*	JN648379
VC1786ICE71_*traG*	100	3570 /3570	VC_6_contig_11	67157..70726	Conjugal transfer mating pair stabilization protein *TraG*	JN648379
VC1786ICE72_*flhC*	100	534 /534	VC_6_contig_11	71248..71781	Flagellar transcriptional activator *FlhC*	JN648379
VC1786ICE73_*flhD*	100	300 /300	VC_6_contig_11	71778..72077	Flagellar transcriptional activator *FlhD*	JN648379
VC1786ICE74	100	549 /549	VC_6_contig_11	72074..72622	Lytic transglycosylase	JN648379
VC1786ICE75	100	663 /663	VC_6_contig_11	72609..73271	Conserved hypothetical protein	JN648379
VC1786ICE76	100	870 /870	VC_6_contig_11	73258..74127	Conserved hypothetical protein	JN648379
VC1786ICE77	100	648 /648	VC_6_contig_11	74552..75199	Putative phage regulatory protein	JN648379
VC1786ICE78	100	195 /195	VC_6_contig_9	168367..168561	Putative DNA-binding protein	JN648379
VC1786ICE79	100	276 /276	VC_6_contig_9	172098..172373	Conserved hypothetical protein	JN648379
VC1786ICE8	100	822 /822	VC_6_contig_24	5232..6053	Conserved hypothetical protein	JN648379
VC1786ICE80	100	566 /594	VC_6_contig_9	175719..176284	Transposase Tn3	JN648379
VC1786ICE81	100	192 /192	VC_6_contig_24	7752..7943	Replication-associated proteinA, *RepA*	JN648379
VC1786ICE83	100	306 /306	VC_6_contig_24	3468..3773	Helix-turn-helix multiple antibiotic resistance	JN648379

					protein, Transcriptional regulator *LysR* family	
VC1786ICE84	100	183 /183	VC_6_contig_24	139..321	Conserved hypothetical protein	JN648379
VC1786ICE85	100	549 /549	VC_6_contig_11	5354..5902	Conserved hypothetical protein	JN648379
VC1786ICE86	100	240 /240	VC_6_contig_11	25363..25602	Conserved hypothetical protein	JN648379
VC1786ICE87	100	327 /327	VC_6_contig_11	48736..49062	Conserved hypothetical protein	JN648379
VC1786ICE88	100	201 /201	VC_6_contig_11	50420..50620	Hypothetical protein	JN648379
VC1786ICE89	100	405 /405	VC_6_contig_11	60068..60472	Glyoxalase/bleomycin resistance protein/ Dioxygenase	JN648379
VC1786ICE90	100	369 /369	VC_6_contig_11	61270..61638	Conserved hypothetical protein	JN648379
VC1786ICE91	100	411 /411	VC_6_contig_11	61703..62113	DNA-binding HTH domain-protein	JN648379
VC1786ICE92	100	546 /546	VC_6_contig_11	62311..62856	Hypothetical protein	JN648379
VC1786ICE93_*dfrA1*	100	474 /474	VC_6_contig_11	62841..63314	Dihydrofolate reductase, resistance to Trimethoprim	JN648379
VC1786ICE94	100	456 /456	VC_6_contig_11	70759..71214	Putative phage membrane protein	JN648379
VC1786ICE95	100	252 /252	VC_6_contig_11	74183..74434	Putative phage regulatory protein	JN648379

**Antibiotic resistance genes**						
***Vibrio cholerae* Gene**	**Identity**	**Query /Template length**	**Contig**	**Position in contig**	**Predicted phenotype**	**Accession number**
VC1786ICE9_*floR*	100	1215 /1215	VC_6_contig_24	3801..5015	Warning	
catB9	100	630 /630	VC_6_contig_36	1574..2203	Chloramphenicol resistance Chloramphenicol acetyl transferase	AF462019

dfrA1	100	474 /474	VC_6_contig_11	62841..63314	Trimethoprim resistance, dihydrofolate reductase, from theICEVchHai1 (VC1786ICE93)	AF221901
gyrA	99.78	453 /453	VC_6_contig_1	356745..357197	Mutated DNA gyrase subunit A. Resistance to Fluoroquinolone	GQ502315
parC	100	210 /210	VC_6_contig_12	70835..71044	Mutated Topoisomerase IV subunit A, resistance to Quinolone	KJ596550
parE	99.72	354 /354	VC_6_contig_12	69869..70222	Mutated Topoisomerase subunit B, resistance to Quinolone	GQ502316
strA	100	804 /804	VC_6_contig_24	1206..2009	Streptomicyn resistance protein *StrA*, from the ICEVchHai1(VC1786ICE 12)	AF321551
strB	100	831 /831	VC_6_contig_24	2015..2845	Streptomycin resistance protein *StrB*, from the ICEVchHai1(VC1786ICE 11)	M28829
sul2	100	816 /816	VC_6_contig_24	330..1145	Sulphonamid resistance dihydropteroate synthase 2, from the ICEVchHai1(VC1786ICE 13)	AY034138

**Serogroup specific genes**						
***Vibrio cholerae* Gene**	**Identity**	**Query /Template length**	**Contig**	**Position in contig**	**Predicted phenotype**	**Accession number**
*rfbV*_O1	100	1233 / 1233	VC_6_contig_14	13785..15017	O-antigen synthesis, O1serogroup	AE003852
**Specie specific gene (*ompW*)**						
***Vibrio cholerae* Gene**	**Identity**	**Query /Template length**	**Contig**	**Position in contig**	**Predicted phenotype**	**Accession number**
ompW	100	416 / 416	VC_6_contig_3	271857..272272	Outer Membrane Protein specific of *Vibrio*	FJ462451

					cholerae	
**Virulence genes**						
***Vibrio cholerae* Gene**	**Identity**	**Query /Template length**	**Contig**	**Position in contig**	**Predicted phenotype**	**Accession number**
VasX	99.74	8853 /10411	VC_6_contig_7	2..8854	Type VI secretion system effector module used by VC against competing eucaryotic and procaryotic cells	KF228943.1
VgrG	98.31	2368 /8442	VC_6_contig_6	625..2989	Type VI secretion system effector module used by VC against competing eucaryotic and procaryotic cells	KC955251.1
als	100	1704 /1704	VC_6_contig_6	168711..170414	Als operon involved in glucose metabolism	vc1590
hlyA	100	217 / 217	VC_6_contig_19	53689..53905	*Vibrio cholerae* hemolysin, causes cytolysis by forming heptameric pores	HQ452880
makA	100	1110 /1110	VC_6_contig_3	256267..257376	Flagella-associated cytotoxin	VCA0883
mshA	100	537 / 537	VC_6_contig_13	28325..28861	Mannose-sensitive hemagglutinin, adhesins/adherence factor	X77217
ompT	99.23	1041 /1035	VC_6_contig_2	446890..447930	pore-forming proteins of the outer membrane, adhesins/adherence factor	AF079766
ompU	99.9	1026 /1026	VC_6_contig_9	138794..139819	Pore-forming proteins of the outer membrane, adhesins/adherence factor	KF434513
rtxA	100	203 / 203	VC_6_contig_6	52431..52633	Multi-functional repeats-in-toxin	AY101181

toxR	98.2	721 / 720	VC_6_contig_1	73544..74264	Transcriptional regulator of *ctx*	HM042639

Biotype specific genes						
Vibrio cholerae **Gene**	**Identity**	**Query /Template length**	**Contig**	**Position in contig**	**Predicted phenotype**	**Accession number**
*tcpA*_et3	100	675 / 675	VC_6_contig_10	44730..45404	Toxin-coregulated pilus specific for El Tor and El Tor Variant biotypes	AF325734

Mobile genetic elements (ICEVchHai1 not included)						
Vibrio cholerae **Gene**	**Identity**	**Query /Template length**	**Contig**	**Position in contig**	**Predicted phenotype**	**Accession number**
No hit found						
**Phage susceptibility elements**						
Vibrio cholerae **Gene**	**Identity**	**Query /Template length**	**Contig**	**Position in contig**	**Predicted phenotype**	**Accession number**
No hit found						

Supplementary Table 3: Mobile genetic elements (MGEs) detected in the isolate

contig	MGEs	**Resistance**
VC 6 contig 24	ISVsa3	*sul2*, *floR*, aph (3')-Ib, aph (6)-Id
VC 6 contig36	0	*catB9*
VC 6 contig11	0	*dfrA1*
VC 6 contig 10	ISVch1	-
VC 6 contig 27	ISVch8	-
VC 6 contig 45	ISVch5	-

**Gene name**	**Phenotype**	**Accession**	**Position in contig**	**Coverage**	**Identity**
sul2	sulfamethoxazole	AY034138	1145-330	100%	100%
floR	florfenicol, chloramphenicol	AF118107	3802-5015	99.92%	98.35%
aph(3")-Ib	streptomycin	AF321551	1206-2009	100%	99.87%
aph(6)-Id	streptomycin	M28829	2009-2845	100%	100%
catB9	chloramphenicol	AF462019	1574-2203	100%	100%
dfrA1	trimethoprim	X00926	62841-63314	100%	100%
